# Unified adult transthoracic echocardiographic report: an expert consensus document of the Egyptian Working Group of Echocardiography

**DOI:** 10.1186/s43044-024-00519-w

**Published:** 2024-07-08

**Authors:** Hala Mahfouz Badran, Adel El Etriby, Azza Elfeky, Gamela Naser

**Affiliations:** 1 Cardiology Department, Menoufia University, 55-El Gish Street, P.O box: 31511, Tanta, Egypt; 2 Cardiology Department, Ain Shams University, Cairo, Egypt; 3 Cardiology Department, Suez Canal University, Ismailia, Egypt

**Keywords:** Echocardiography, Echocardiographic report, Cardiac function, Standardized reporting, Egypt

## Abstract

**Background:**

Physicians face complexity in interpreting the results of echocardiography (ECHO) due to the variability across ECHO laboratories. Many international organizations published reports to reduce the inter-variability in ECHO reporting. However, with the evolution of imaging modalities, significant improvements in ECHO reporting are essential to eliminate any previous discrepancies. The Egyptian Working Group of Echocardiography (EEWG) aimed to prepare a standardized, updated, simple, and comprehensive ECHO reporting in Egypt to offer consistency, guarantee that all the crucial features are fulfilled, and ease practitioners' communication to maximize clinical decision-making.

**Main text:**

Relevant articles were retrieved and reviewed to explore the current state of TTE reporting practices, existing guidelines, and challenges faced by physicians in interpreting TTE results. Identified gaps and areas for improvement were then employed to establish the outline for the standardization approach. This report addresses crucial components such as demographic data, measurements, and interpretative summaries. It emphasizes left ventricle measurements and systolic function assessment, incorporating advanced techniques like speckle tracking and three-dimensional imaging. The significance of evaluating diastolic function, examining the right ventricle, and assessing valves, pericardium, and aorta are also discussed.

**Conclusion:**

The current consensus goals to streamline communication among practitioners contribute to a more unified approach to interpreting ECHO results. Our initiative marks a significant step forward in enhancing the standardization and quality of ECHO reporting in Egypt. By introducing this report and encouraging continuous learning, the working group aims to raise the overall reporting quality and facilitate interpretation across diverse echocardiographic settings. This concerted effort improves patient care by ensuring consistency, accuracy, and relevance in interpreting echocardiographic findings.

## Background

Transthoracic echocardiography (TTE) is a dynamically evolving, broadly available, safe, and easy-to-apply imaging tool for the quantitative and qualitative investigation of heart structure and function [1]. Utilizing two-dimensional (2D) echocardiography conjunctively with other imaging techniques, for instance, real-time three-dimensional echocardiography (3DE) and speckle tracking echocardiography, significantly widened the clinical application of echocardiography (ECHO) imaging regarding cardiac anatomy and function [2].

However, in some conditions, physicians face complexity in interpreting the results of TTE due to the variability across ECHO laboratories resulting from the variant contents, reporting forms, and terminologies, in addition to the unavailability of some advanced modalities in many centers. Accordingly, physicians exert more effort to extract clinically useful information from variable TTE reports. As a result, several guides were published to help physicians recognize ECHO findings promptly [3, 4].

Therefore, significant improvements in TTE reporting modality are required. Several international efforts were introduced to decrease the inter-variability in reporting TTE findings. In 2002, the American Society of Echocardiography (ASE) issued its first endorsements for a standardized structured report for adult TTE to guide echocardiographers on which measurements and descriptive data are reported in the TTE form. This is to enhance the reporting quality using definitions of the essential measurement items and reports to be included [5]. In 2010, the ASE, EACVI (the European Association of Cardiovascular Imaging), and others started an initiative to regulate strain imaging [6].

Afterward, after the evolution of TTE modalities, ASE and EACVI continued to update their previously published guidelines regarding the recommendations and average reference values, considering the elimination of any previous discrepancies. The prepared document standardized a methodology for quantifying cardiac chambers and establishing reference values to offer uniformity and facilitate practitioners' communication [7].

Finally, the EACVI issued in 2017 an expert consensus document to standardize modern TTE reporting and update the current ASE/EACVI cardiac chamber quantification, diastolic function, and valvular heart disease (VHD) statements to meet the expectations of echocardiographers and referring physicians [8].

In the era of multimodality imaging, standardized, simple, and comprehensive, TTE reporting is mandatory to guarantee that all the relevant aspects are fulfilled in a TTE report to inform clinical decision-making maximally. This will help avoid inadequate fulfillment of the clinical question, the inconsistency between the clinical assessment and the TTE results, and inter-measurement variability.

The Egyptian Working Group of Echocardiography (EEWG) aims to promote knowledge and raise the quality standards of ECHO reporting in adults in all centers and laboratories all over Egypt. We are attentive to allowing this model of the ECHO report to different echocardiographic working groups, societies of cardiology and committees, boards, or societies of echocardiography, and not the establishment of rigid learning criteria. Launching this unified ECHO report encourages learning, raises the quality of reporting, and facilitates TTE report interpretation among the relevant stakeholders irrespective of accreditation level, experience, or subspecialty.

## Methodology

This standardized reporting form was developed by the EEWG, which convened a multidisciplinary team of experts comprising echocardiographers, cardiologists, and researchers. Relevant articles were retrieved and reviewed to explore the current state of TTE reporting practices, existing guidelines, and challenges faced by physicians in interpreting TTE results. Identified gaps and areas for improvement were then employed to establish the outline for the standardization approach. The EEWG team also held multiple meetings and discussions to critically evaluate the existing guidelines and reporting formats issued by international organizations. After revising all available documents, data, and research workup, an updated, comprehensive, and simple format was developed to unify the reporting aspects of ECHO findings in Egypt.

## Component *of ECHO* report

**Fig. 1:**
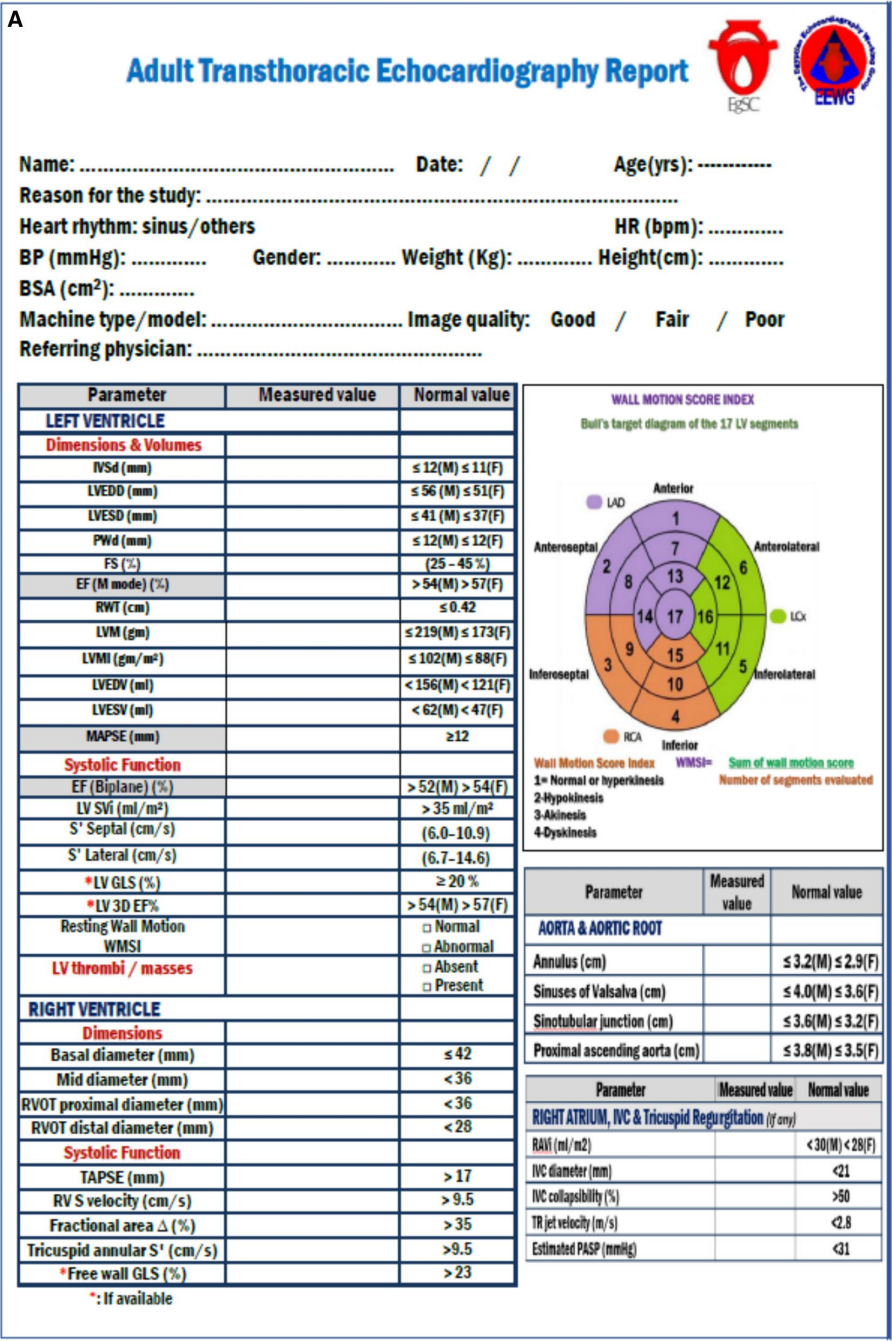

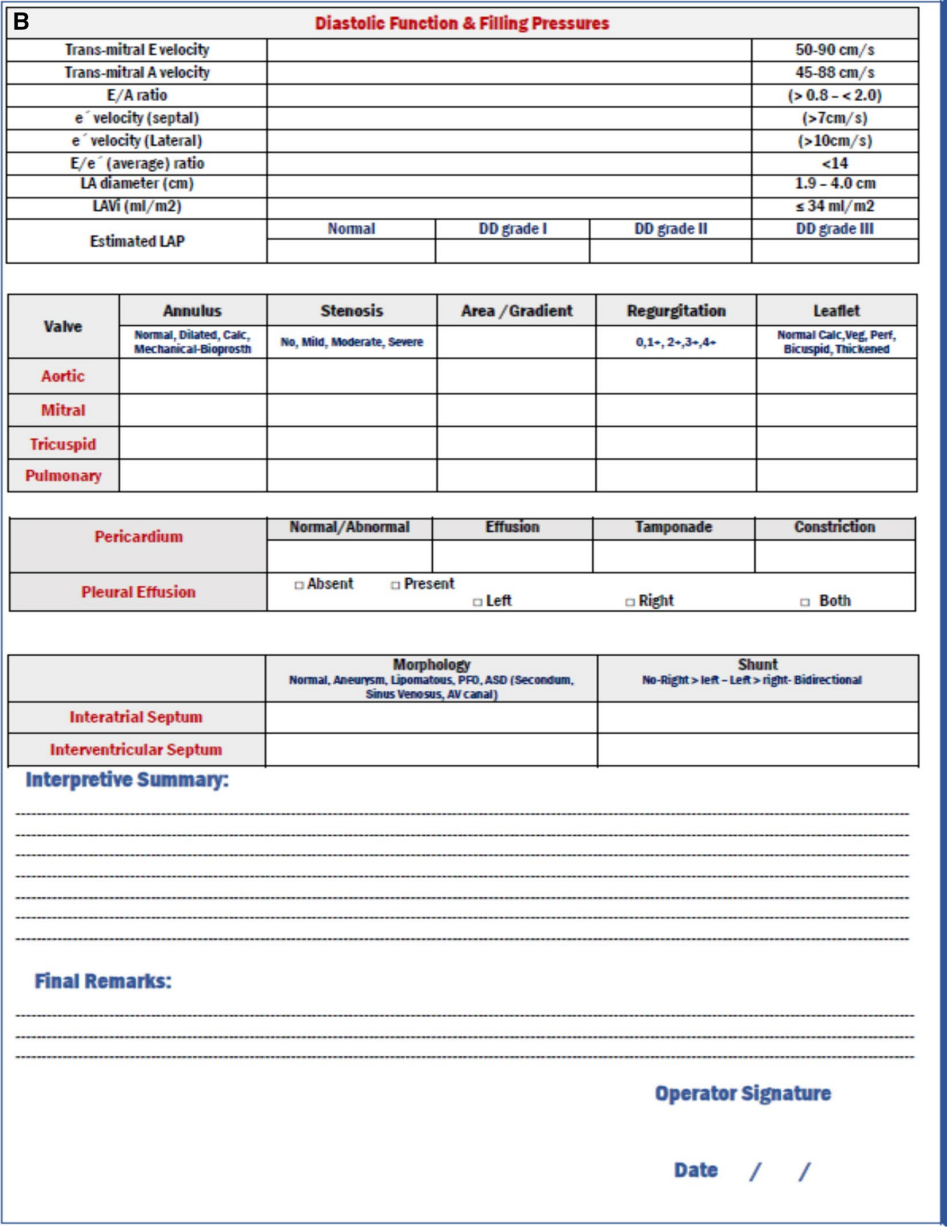
ECHO report format (**A**, **B**)

The ECHO report format is composed of three sections (Fig. 1A, B):

### Demographic data

Patient's data and identifying information include patient's name, date, age, gender (male or female), and patient's height and weight, calculated body surface area (BSA) [some variables should be indexed to body surface like left ventricular mass index (LVMI) and left atrium volume index (LAVI)]. In addition, the reason for the ECHO order (e.g., preoperative evaluation of LV function, evaluation of VHDs, follow-up following cardiac surgery), heart rhythm (sinus or atrial fibrillation (AF)), heart rate (HR), and systolic and diastolic blood pressure is included.

Also, stating the ECHO technique type, either vendor or model, is mandatory. The advanced ECHO machines show inter-vendor and software inconsistency, especially in developed ECHO criteria currently in use, for example, LV strain (longitudinal, circumferential, radial, and other variables, principally those obtainable by tissue Doppler imaging (TDI)) [9].

### Measurements section

A measurement section includes LV and right ventricular (RV) dimensions and functions (using different modalities like strain and TDI), the aorta and aortic root diameters, assessment of left (LA) and right atria (RA), LV filling pressure, inferior vena cava (IVC) diameter and collapsibility index. In addition, the section includes an assessment of the cardiac valve's morphology and structure, as well as functions and a description of the pericardium and atrial and ventricular septae.

### Interpretative summary

At the end of the ECHO report, a summary is included, objectively linked to the questions derived from the ECHO examination and highlighting the fundamental ECHO findings. This summary should be clear, comprehensive, and reasonable to any physician. Answering any limitation or factor affecting the conclusions should be reported.

It usually comprises statements to answer the referring physician's questions, confirm unusual results, and describe the most relevant data and variations and similarities of the present ECHO versus prior studies. These descriptive statements usually focus on the main findings and signify the main probable findings, balancing the need for a brief and complete response to the request raised by the patient and referring physician. ECHO laboratories may adopt these abbreviated statements to compose sentences for this part of the report.

## Contents *of ECHO* report

### Left ventricle measurements

#### Structural measurements

The ECHO report should contain the measurement of LV internal dimensions, end-systolic diameter (ESD), and end-diastolic diameter (EDD). This is combined with the described 2D-derived LV volume measures, such as end-diastolic volume (EDV) and end-systolic volume (ESV) indexed for BSA, end-diastolic septal wall thickness (IVSd), and posterior wall thickness (PWd). The end-diastole refers to the first frame directly after mitral valve closure or as the peak of the R-wave on the electrocardiogram (ECG).

Measurable values of LV mass and relative wall thickness (RWT) may follow LV structure and geometry measurements. These values are obtained from LV internal diameters and wall thicknesses at end-diastole, determining LV hypertrophy and concentric or eccentric remodeling, respectively [7].

LV mass assessment is carried out by utilizing the area–length or truncated ellipsoid method. By utilizing one of these 2D methods, the average LVMI is identified as ≤ 88 g/m2 in females and ≤ 102 g/m^2^ in males [10]. In addition, relative wall thickness is determined as (two × posterior wall thickness) / (LV internal diameter at end-diastole). If the RWT is above 0.42, it states concentric remodeling, while a value less than or equal to 0.42 states eccentric remodeling.

#### Functional measurements

##### Systolic function

Even with its limitations, LV ejection fraction (LVEF) is one of the commonly tested measures in ECHO. LVEF is still considered the most fundamental causative factor for cardiac outcomes in various clinical conditions [11]. There are numerous quantitative approaches to EF measures in ECHO. Fractional shortening (FS) and Teichholz's method for estimation of EF from M-mode imaging have been displaced by LV volume measurements and EF% in apical 4 and 2 chamber views by applying Simpson's biplane methodology [12].

LV regional systolic function must be reported by visual assessment of 17 (or 18 or 16) segments of the wall motion score index (WMSI), which is inversely correlated with LVEF. Reporting LV segments indicates coronary perfusion territories and allows consistent correlation with more non-invasive imaging techniques. A model with 17 segments (with the apical cap at the top of the four different apical segments: septal, inferior, lateral, and anterior) must be interrogated.

##### Mitral annular plane systolic excursion (MAPSE)

**Fig. 2:**
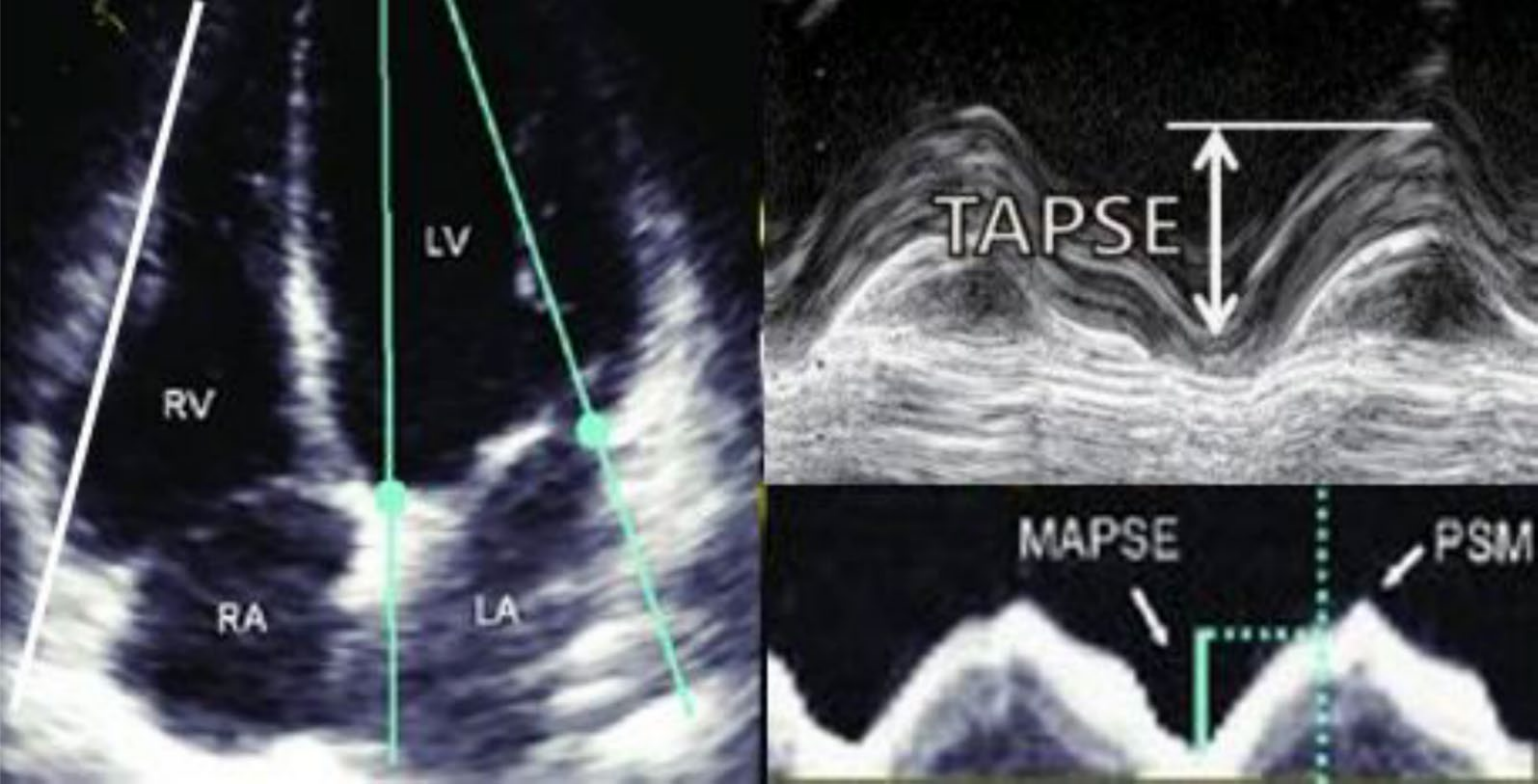
Annulus plane systolic excursion, mitral (MAPSE), and tricuspid annular plane systolic excursion (TAPSE)

The mitral annular plane systolic excursion (MAPSE) is measured with an M-mode line placed through the mitral annulus (Fig. 2). It involves the tissue–blood border for easy recognition of the motion of the annulus. The end-diastolic position of the annulus, quantified at the tip of the QRS complex, refers to the trough of the motion. At the maximal systolic excursion point, the peak is measured. The mean MAPSE value (averaged from septal, lateral, anterior, and inferior MAPSE) could be quantified [13].

##### Tissue Doppler of the mitral annulus

The tissue Doppler imaging technique assesses the longitudinal movement velocities of the lateral and medial mitral annulus [10, 14, 15]. The angle of interrogation should be parallel to the Doppler beam. Tissue Doppler signals are adjusted by using a sample volume of 5–10 mm to completely capture annular motion [10] to improve visualization of the peak annular velocities, decrease the velocity scale to duplicate Doppler signal display [12], and set the sweep speed at 100 mm/sec. Velocity waveforms are created s' for systole, e' for early diastole, and a' for atrial contraction. Establishment of mean value of the three velocities would be made from averaged lateral and medial velocities of all three components. The averaged e' is used for quantification of the E/e' ratio. Some measurements are applied for the lateral tricuspid annulus [13, 16]. The utmost critical quantification for the right ventricle is the s' velocity, which correlates with global RV systolic function alternative measures [13].

##### Three-dimensional (3D) LV function

Presenting 3DE LV volumes and EF in the report that are not based on geometric assumptions is valuable (if available). This will increase precision and reproducibility in patients with acceptable imaging quality [17]. The LV position is also essential for the entire chamber to be seen in the volume set at highest possible frame rate. Numerous softwares provide semiautomated algorithms for volumetric calculation. This technique possibly provides the best link of ECHO-derived volumes to reference standards when there is high image quality [18].

##### LV strain

**Fig. 3:**
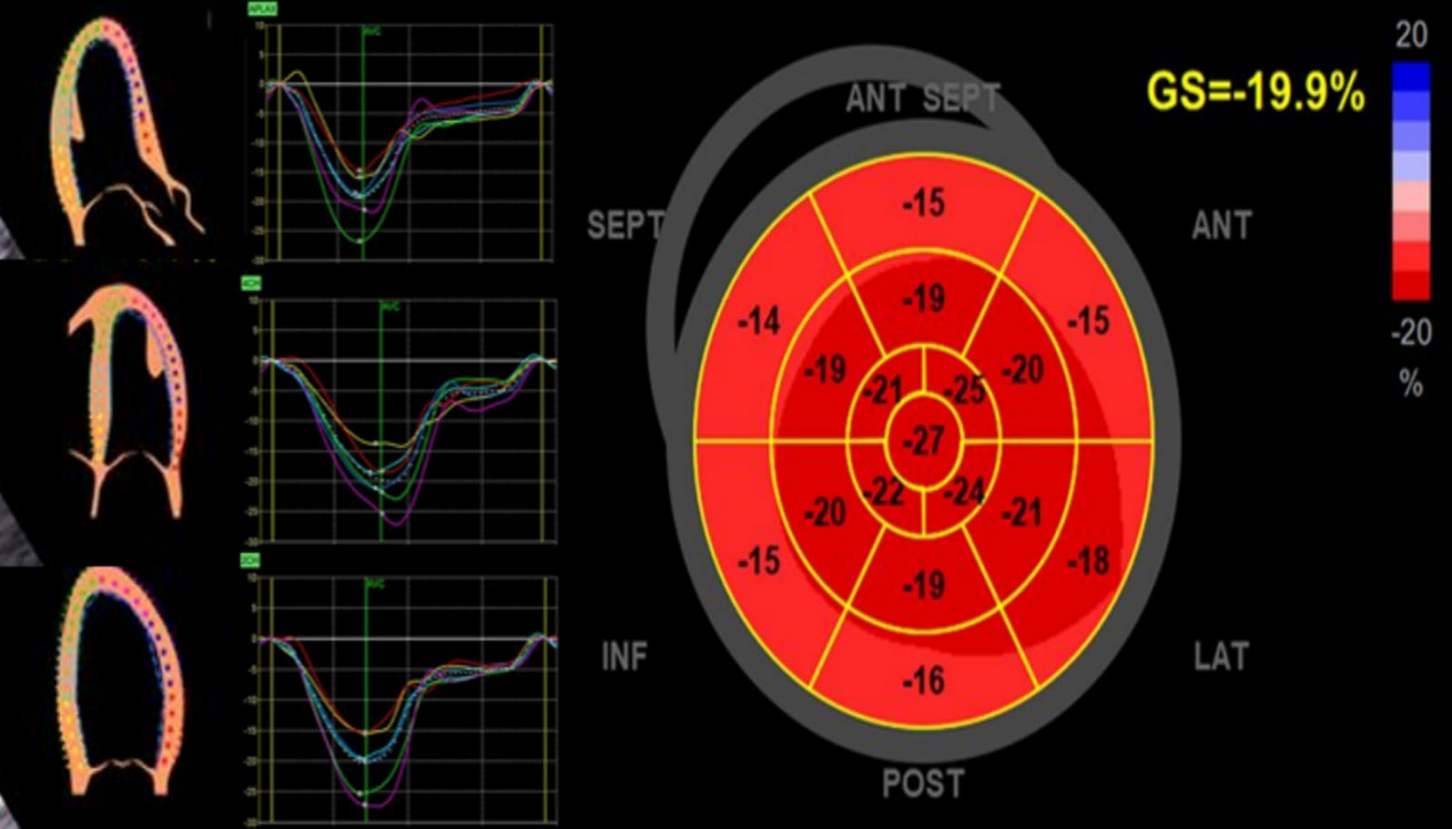
Bull's eye map of peak segmental and global longitudinal strain of left ventricle

Global longitudinal strain (GLS) using speckle tracking is advised to be included in the ECHO report to present LV longitudinal function quantitative analysis (Fig. 3). GLS accurately and promptly detects subclinical changes in LV longitudinal function earlier to LVEF disturbance [2, 6]. Also, GLS is increasingly used and considered a convenient, reproducible technique [19]. It also provides an incremental predictive value for identifying LV function at rest [2]. There is no definite cutoff value due to vendor variability in type and model. However, a range of 18% to -22% could be considered normal. Stroke volume and cardiac index are essential, particularly in assessing VHD [20].

##### Diastolic function and LV filling pressure

o ***Left atrium (LA)***

Bidimensional linear anterior–posterior quantifications of the LA are favorite to be reported in the ECHO report. This can be done using M-mode ECHO. LA is calculated at end-systole utilizing a leading edge–to–leading edge technique [7]. For the 2DE method, the caliper is positioned at the level of the sinus of the valsalva of the aortic root and expanded to the leading edge of the posterior LA wall perpendicular to the presumed LA long axis [7]. For M-mode, the cursor is oriented perpendicular to the aortic root and LA at the level of the aortic sinuses. The measurement caliper is placed at the leading edge of the posterior wall of the aortic sinus and expanded to the leading edge of the posterior LA wall [7].

o ***LA Volume***

**Fig. 4:**
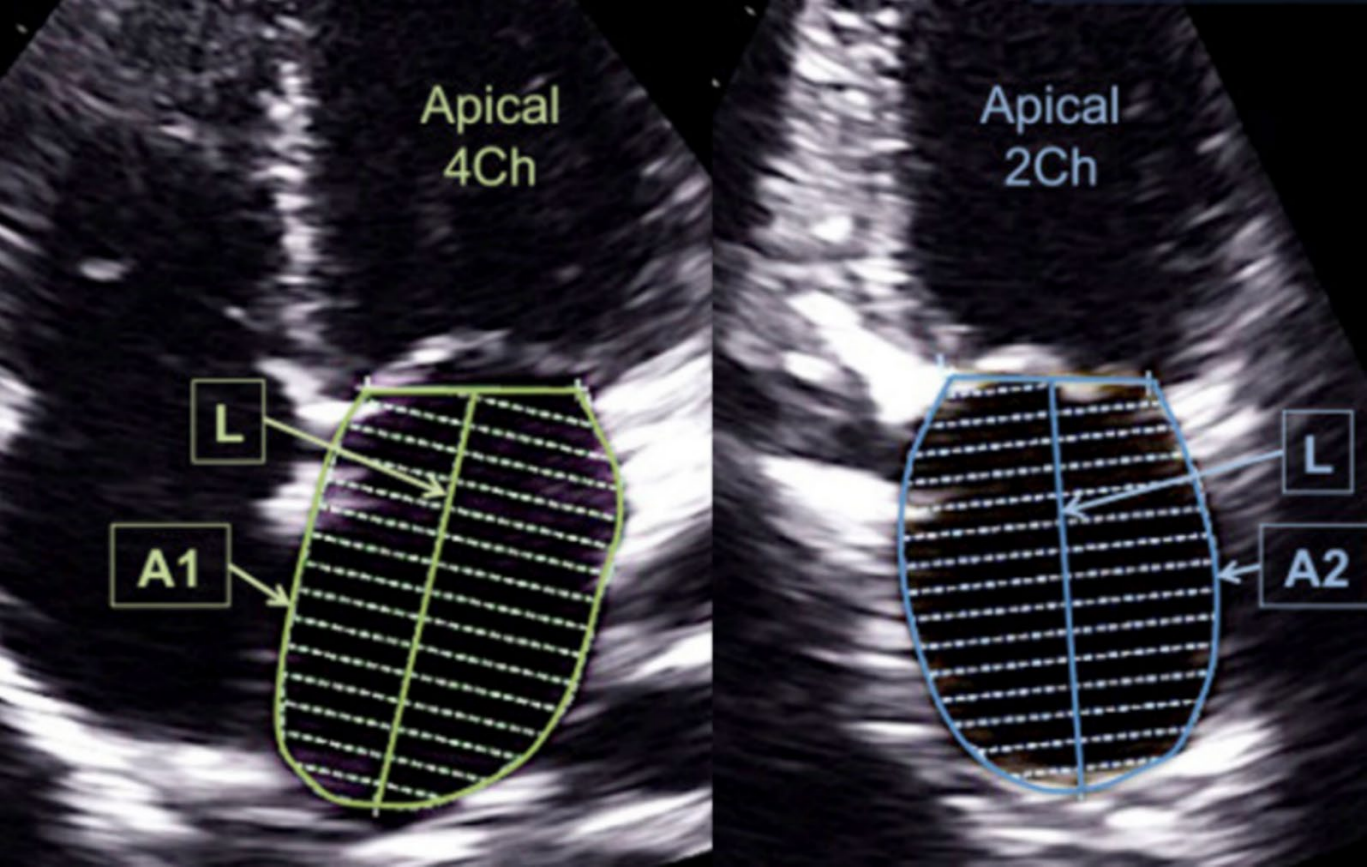
2D ECHO assessment of LA maximal volume by tracing of left atrium endocardium in apical four-chamber and two-chamber view

2D LA images are acquired in the ECHO report and adjusted for volume quantification [7]. Initially, maximum volume at end-systole is determined (Fig. 4). Then, LA endocardial borders in the A4C and A2C views are traced. LA tracing begins with one aspect of the mitral annulus corner and ends by reaching the aspect of the annulus to the opposite side. The atrial appendage and pulmonary veins would not be involved in the tracing. The length of the LA must be measured in both the A4C and A2C views. This length is measured from the mitral annulus to the inner edge of the furthest extent of the traced superior LA wall at the approximate midpoint. The long-axis lengths should be within 5 mm of each other. If they are not, the apical images should be reevaluated [21]. Most ECHO machines automatically calculate LA biplane volume using area length and biplane disk summation. With the area-length method, the shorter length obtained (in the two- or four-chamber view) would be used for measuring the LA volume (Fig. 3) [7, 21]. Using the method of disks, the longer of the two lengths should be used. Disks are the preferred method for calculating LA volume, as they involve less assumptions about the shape of the LA [7]. As the volume calculation of LA changes with different techniques, it is necessary to utilize the same technique [22].

Conventional ECHO offers valued data to evaluate LV diastolic function and estimate LV filling pressure (Table 1). It also enhances both the diagnostic and prognostic worth. Besides clinical features, ECHO can support identifying heart failure with preserved EF (HFpEF) and the need for advanced testing. Sometimes, ECHO alone is inadequate for diagnosing HFpEF, and several times, invasive hemodynamic exercise testing and more advanced testing are essential.

**Table 1:** Diastolic function assessment using conventional ECHO parameters

Diastolic dysfunction (DD): estimation of LAP in normal/ reduced EF		
3 Criteria	Measured	Value
Average E\e' TR velocity LA Vol. index		> 14 > 2.8 m/s > 34 ml/m2
E Velocity E/A ratio		
**E < 50cm/s and E/A ≤ 0.8**		
Furthermore, 2 of the three criteria are negative		Normal LAP
2 of 2 available criteria are negative		Grade I DD
If the patient is symptomatic, consider CAD or perform diastolic stress		
**E/A ≥ 2**		Increased LAP Grade III DD
**E > 50 cm/s and E/A ≤ 0.8 Or E/A > 0.8—< 2** And two of the three criteria are positive Or two of the two available are positive		Increased LAP Grade II DD
One negative and one positive of the two available Or only of three criteria are available		Indeterminant LAP Intermediate DD
Pulmonary vein S/D ratio < 1 can conclude elevated LAP (If EF < 50%)		

CAD: coronary artery disease, DD: diastolic dysfunction, e′: mitral annular early diastolic tissue velocity, E/A: early diastolic to late diastolic mitral inflow velocities, E/e′: ratio of E to e', EF: ejection fraction, LA: left atrium, LAP: left arterial pressure, TR: tricuspid regurgitation

An integration of different diastolic function parameters is mandatory to diagnose HFpEF including mitral flow velocities, the ratio of early diastolic to late diastolic mitral inflow velocities (E/A), deceleration time of E wave, mitral annular early diastolic tissue velocity (e′), ratio of E to e' (E/e′), pulmonary venous velocities, tricuspid regurgitation (TR) velocity jet, and LA maximum volume index (Table 1) [23, 24].

### Right ventricle measurement

#### RV linear dimensions

In the RV-focused apical 4C view, the RV linear longitudinal end-diastolic dimension is calculated by drawing a line from the midpoint of the tricuspid annulus to the interface of the compacted myocardium at the chamber apex. Diameter calculations include the maximum transverse diameter in the basal third of the right ventricle at end-diastole and the mid-cavity linear dimension midway between the maximal basal diameter and apex. The mid-cavity diameter measurement is made at the level of the papillary muscles at end-diastole [7, 13].

#### RV area

**Fig. 5:**
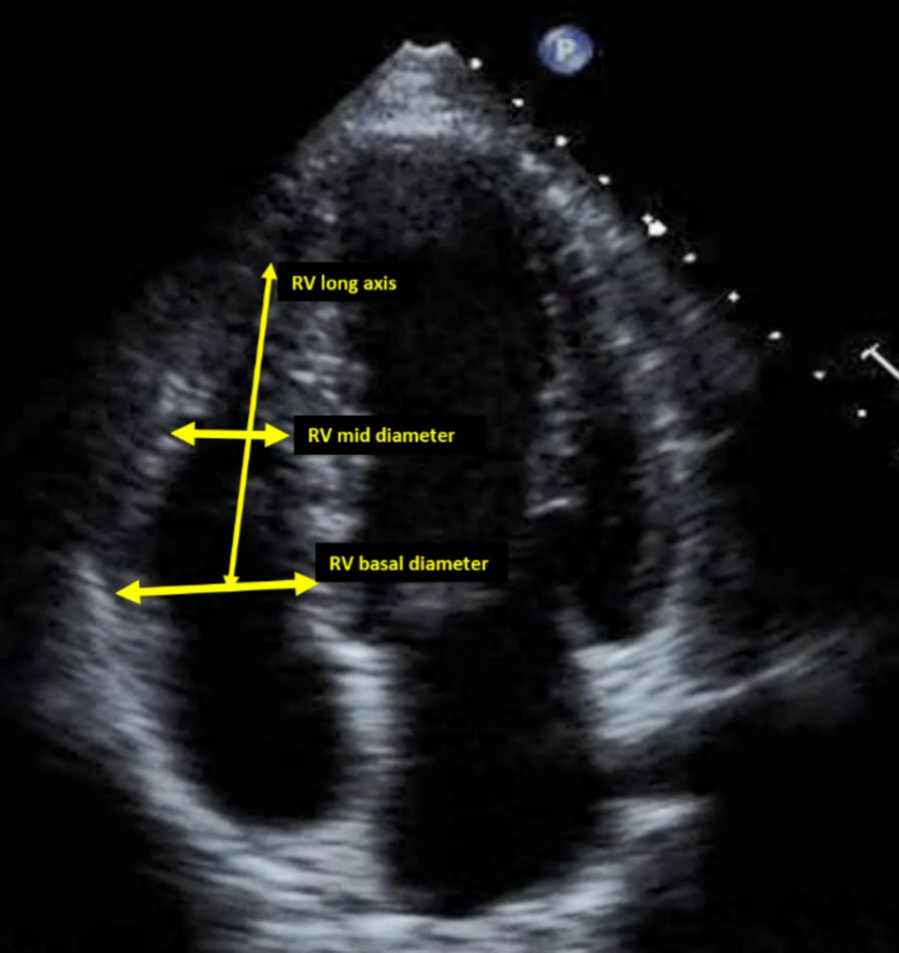
RV-focused apical four-chamber image showing measurement of RV long-axis and basal and mid-minor axis diameters

In the RV-focused (Fig. 5) apical 4C view, the RV area is calculated by tracing the compacted muscle blood–endocardial tissue border of the right and noncoronary leaflet insertion points at the maximal opening of the valve near mid-systole.

#### Tricuspid annular plane systolic excursion (TAPSE)

TAPSE measures longitudinal systolic function of the RV [25, 26]. It is measured in the apical 4C view [13, 14]. An M-mode cursor should be aligned along the RV free wall as perpendicular to the lateral tricuspid annulus as possible (parallel to the movement of the TV annulus). The TV annulus region of interest (ROI) should be verified as much as possible without removing relation of the anatomic structures. The distance moved by the leading edge of the annulus from end-diastole toward the apex at end-systole is computed.

### Tricuspid valve

Tricuspid valve inflow velocity is recorded through at least one respiratory cycle with pulsed wave Doppler. The TV inflow is diastolic with two distinct waves: the first in early diastole (E velocity) and the second at late diastole during atrial contraction (A velocity). TV inflow velocity is better assessed in apical 4C view and/or RV-focused views depending on the best Doppler angle interrogation.

### Aorta

**Fig. 6:**
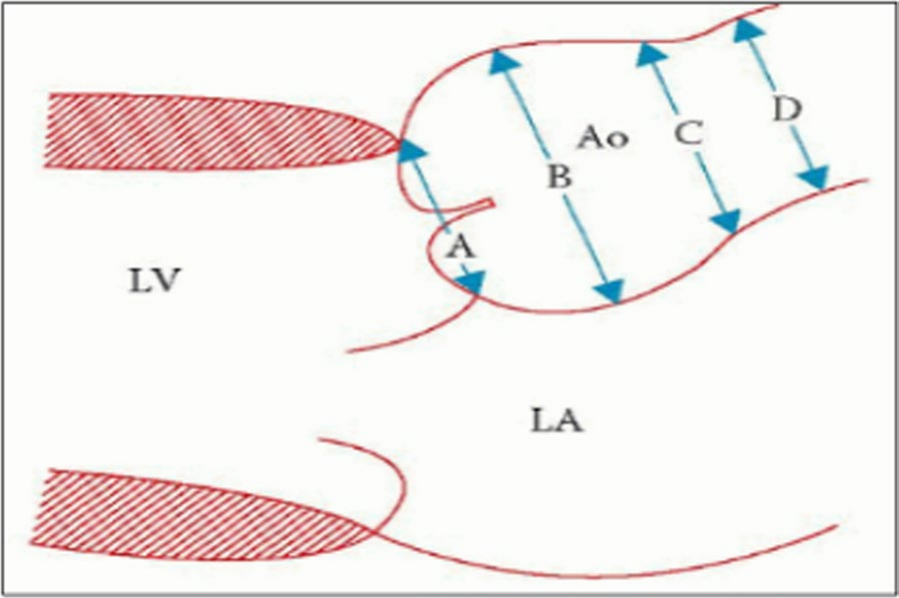
Aortic dimensions estimated from parasternal long-axis view. Aortic diameters consisting of aortic A: annulus, B: aortic root (maximal diameter at the sinus of Valsalva), C: sinotubular junction, and D: proximal ascending aorta

Despite this, TTE does not permit a whole evaluation of the aorta. It detects any change of specific sections since the proximal aorta is the frequent dilation area [27]. Aortic diameters are estimated from the parasternal long-axis view, and the zoomed view is advised (Fig. 6). Mid-systolic measurements of the LV outflow tract and aortic annulus are recommended. As seen in Fig. 6, aortic diameters consisting of aortic annulus, aortic root (maximal diameter at the sinus of Valsalva), sinotubular junction, and proximal ascending aorta are mandatory to provide a thorough evaluation of thoracic aortic size. The aortic annulus can be estimated by inner edge-to-inner edge, while all other measurements can be estimated using the leading edge-to-leading edge convention at end-diastole [7]. Entirely these dimensions must be measured by 2D rather than M-mode ECHO and indexed for BSA [7, 27]. Imaging and measurements of the aortic arch through the suprasternal view are highly advised when plaque, thrombus, or dissection is presumed [28]. A difference of less than three millimeters can happen due to retest variation, regardless of the imaging technique. Thus, when making serial assessments, such differences must be taken with caution.

### Inferior vena cava (IVC)

The IVC diameter is calculated using M-mode from the subcostal (SC) long-axis view [29], on supine position. The measurement would be made 1–2 cm proximal to the IVC insertion into the right atrium at its maximum dimension, commonly during expiration [7, 13]. An adequately long recording should be made for any changes seen in IVC diameter during the respiratory cycle. The IVC diameter is calculated at rest and during normal respiration, and the IVC collapsibility index is calculated from the maximum diameter on expiration—(minimum diameter on inspiration/maximum diameter on expiration). If the central venous pressure is normal, the diameter of the IVC typically collapses more than 50% of its expiration diameter. If not, or a less collapse is seen, the patient is asked to perform a rapid inspiratory "sniff" to force a more significant change in intrathoracic pressure and record IVC motion a second time. Consider that the M-mode cursor perpendicularly transects the IVC so that the diameter is not incorrectly overvalued. Imaging the IVC in the short axis during the sniff maneuver can help determine if the IVC translates out of the imaging plane during inspiration. This information is combined with the IVC diameter to estimate proper atrial pressure.

### Valve assessment

The primary aim of ECHO is to specify the etiology, mechanism, severity, and effect of the regurgitant lesion on the chamber remodeling. Table 2 demonstrates assessment of the aortic, mitral, tricuspid, and pulmonary valve stenosis and regurgitation. In VHDs, it is crucial to give sufficient data in the ECHO report on the type, degree of valvular dysfunction, and the hemodynamic burden provoked by the valve defect to enhance diagnosis, stratify prognosis, and address management.

**Table 2:**
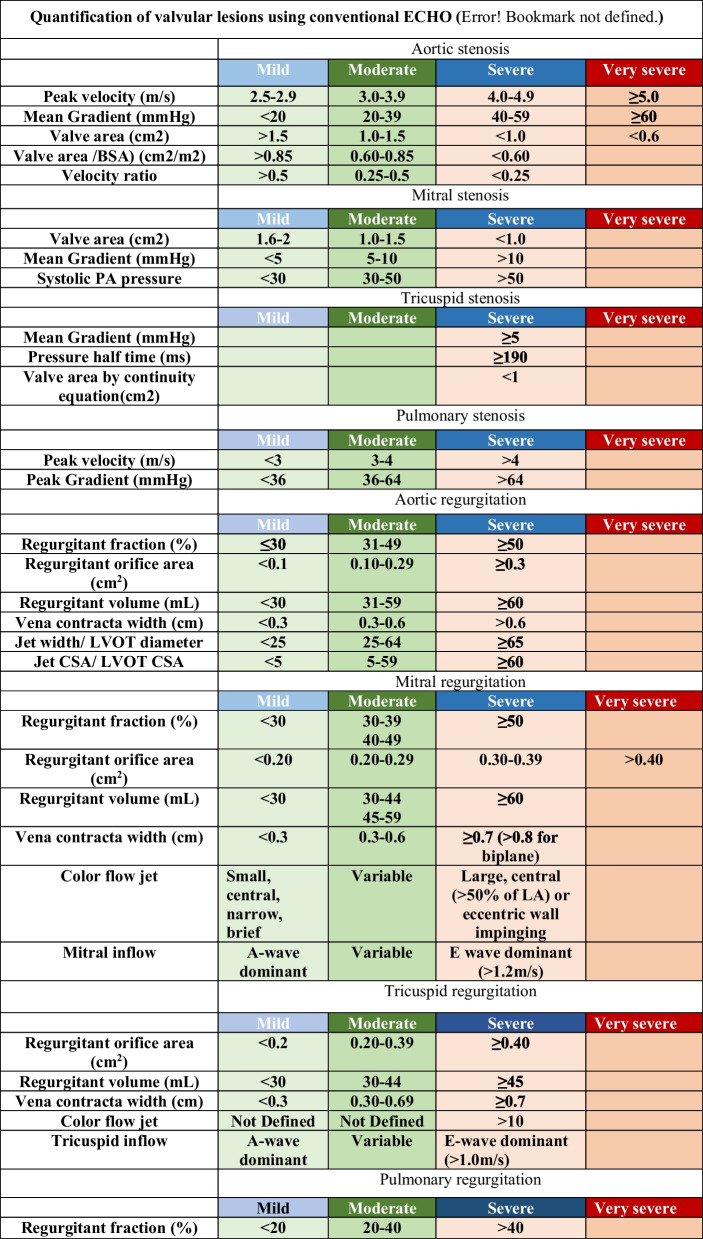
Quantification of valvular lesions using conventional ECHO (8)

Presenting left and right cardiac valves in the report should consider the type of valve dysfunction. 2D TTE clearly distinguishes severe regurgitation from a minor leak in regurgitant valve disease. The description of valve morphology is necessary to pinpoint the etiology (primary/organic or secondary/functional).

The severity of valve defects is examined based on a combination of 2D and Doppler-derived features [23, 24, 30]. For stenotic lesions, valve area and transvalvular gradients are the main parameters (Table 2), and both techniques should be used simultaneously for precise analysis.

The severity of regurgitant lesions relies on both qualitative and quantitative variables, including jet size (planimetry of jet area, jet width, or vena contracta), effective regurgitant orifice area, regurgitant volume and fraction, and the impact of regurgitation on upstream and downstream cardiac [23, 24] is required when analyzing Doppler-based measurements because these measurements are impacted by hemodynamics and may lead to erroneous interpretation when examined separately. Valve gradients are generally exaggerated in the presence of tachycardia and increased cardiac output state. Meanwhile, regurgitant lesions are suppressed when systolic blood pressure is decreased.

In addition, cautious assessment of the valve morphology is beneficial to identify the severity of the valve defects. The ECHO report should comment on the valve annulus (normal, dilated- calcified- mechanical, bioprosthetic) and valve leaflets (normal, calcified, vegetation, perforation, bicuspid, or thickened).

After a substantial valve lesion is observed, a comprehensive assessment is essential. The assessment includes valve morphology, LV systolic function, intracardiac hemodynamics, coexisting valvular and non-valvular pathologies, etc. Balloon mitral valvotomy in rheumatic mitral stenosis, mitral valve repair or replacement in mitral regurgitation, concomitant aortic root replacement in a patient with aortic valve disease, and others are substantially affected by the valve morphology [28, 31].

Aortic stenosis: The primary hemodynamic parameters recommended for clinical evaluation of AS severity are: the peak aortic velocity, the mean gradient, the valve area and its indexed value, and the ratio between jet maximum velocity (V) of LVOT and aortic valve (AV): VR = V_LVOT_/V_AV_.

Mitral stenosis: Mitral valve area is estimated by planimetry of mitral valve orifice including opened commissures, on a parasternal short-axis view. For measuring the mitral valve gradient, the continuous wave Doppler (CVD) is preferred using the apical window in most cases as it allows for parallel alignment of the ultra sound beam and mitral inflow.

Tricuspid stenosis: Careful examination of valve thickening and/or calcification, restricted mobility with diastolic doming, reduced leaflet separation at peak opening, and right atrial enlargement are required.

The hallmark of a stenotic valve is an increase in transvalvular velocity recorded by CWD. The mean pressure gradient derived using the 4v2 equation is lower in tricuspid than in MS, usually ranging between 2 and 10 mmHg, and averaging around 5 mmHg. Higher gradients may be seen with combined stenosis and regurgitation.

Pulmonary stenosis: Calculation of pulmonic valve area by planimetry is not probable since the required image plane is in overall unavailable. CWD is usually applied in parasternal short-axis view.

Aortic regurgitation: Parasternal long- and short-axis views in addition to apical view are essential in evaluating the origin of the regurgitatnt jet and its semiquantitative characteristics. It is important to visualize the three components of the color jet (flow convergence, VC width, and jet area) for a better assessment of the origin and direction of the jet and its overall severity (Table 2).

Mitral regurgitation: In primary MR, an intrinsic abnormality of the leaflets causes the MR, whereas secondary MR results from distortion of the MV apparatus due to LV and/or LA remodeling. Measurement of regurgitant fraction, regurgitant volume, regurgitant orifice area, and VC width is essential in addition to color flow jet.

Tricuspid regurgitation: The parasternal RV inflow view will always image the anterior leaflet in the near field, but in the far field, the leaflet may be the septal or the posterior leaflet. On short axis, the leaflet adjacent to the aorta is either the septal or anterior leaflet, and the leaflet adjacent to the RV free wall is usually the posterior leaflet. Like MR parameters, regurgitant fraction, regurgitant volume, regurgitant orifice area, and VC width are used to assess the severity of TR.

Pulmonary regurgitation: The proximal jet width (VC) is probably the most widely used semiqualitative color Doppler method. The VC width of the PR is commonly expressed as a ratio relative to the PV annulus diameter. A ratio of > 0.5 is correlated with severe PR measured by CMR.

### Pericardium

Standard pericardial thickness is ≤ 2 mm. Pericardial effusion (PE) is seen as an ECHO-free space between the two layers of the pericardium. The volume of PE has not been consistent with the patient's clinical symptoms. PE in adults is classified according to size, including trivial (seen only in systole), mild (< 10 mm), moderate (10–20 mm), and severe (> 20 mm) [32]. The frequent, simple reproducible view is the parasternal long axis, but it remains important to match with all standard views, like the parasternal short axis and the 4Cview. A subcostal view is also routinely utilized; nonetheless, it is also essential not to overestimate the effusion size affected by the probe's angle. At the end-diastole, ECHO-free space between both pericardial layers can be predicted along the posterior wall of the LV. M-mode ECHO in the parasternal long axis reveals an ECHO-free space between the visceral and the parietal pericardium. This sign is only noticed in systole in case of mild effusions and during the complete cycle when the effusion is at least moderate.

If cardiac tamponade is suspected, the data required should assess (1) quantity and quality of pericardial fluid; (2) collapsibility of cardiac chambers; (3) diastolic ventricular size variability with respiratory cycle; (4) septal "bounce"; (5) collapsibility of the IVC; respiratory variation of flow patterns through and semilunar valves; and (6) hepatic and pulmonary veins flow patterns. The first five signs are easily obtained with both 2DE and M-mode. Assessment of flow pattern variability will require Doppler evaluation.

While a rigid or thickened pericardium is the anatomic substrate responsible for constrictive physiology, a thickened pericardium may appear without constrictive features, especially in patients who have had thoracic radiation therapy or open-heart surgery.

## Final remarks

All ECHO findings should be considered concerning the clinical scenario of the patient. Additional investigation using transesophageal ECHO, cardiac computerized tomography, magnetic resonance imaging, nuclear imaging, and coronary angiography could be recommended if diagnostic uncertainty continues.

Medical writing and editorial support were provided by Novartis Egypt, with no influence on the content of the manuscript.

## Data Availability

Data sharing is not applicable to this article as no datasets were generated or analyzed during the current study.

## Abbreviations

2D: Two-dimensional
3D: Three-dimensional
3DE: 3D echocardiography
AF: Atrial fibrillation
ASE: American Society of Echocardiography
BSA: Body surface area
CAD: Coronary artery disease
DD: Diastolic dysfunction
EACVI: European Association of Cardiovascular Imaging
e′: Mitral annular early diastolic tissue velocity
E/A: Early diastolic to late diastolic mitral inflow velocities
E/e′: The ratio of E to E'
EEWG: Egyptian Working Group of Echocardiography
ECG: Electrocardiogram
ECHO: Echocardiogram
EDD: End-diastolic diameter
EDV: End-diastolic volume
EF: Ejection fraction
ESD: End-systolic diameter
ESV: End-systolic volume
FS: Fractional shortening
HFpEF: Heart failure with preserved EF
HR: Heart rate
GLS: Global longitudinal strain
IVC: Inferior vena cava
IVSd: End-diastolic septal wall thickness
LA: Left atrium
LAP: Left arterial pressure
LAVI: Left atrium volume index
LV: Left ventricular
LVEF: LV ejection fraction
MAPSE: Mitral annular plane systolic excursion
PWd: Posterior wall thickness
RA: Right atrium
ROI: Region of interest
RV: Left atrium
SC: Subcostal
WMSI: Segments wall motion score index
TAPSE: Tricuspid annular plane systolic excursion
TDI: Tissue Doppler imaging
TR: Tricuspid regurgitation
TTE: Transthoracic echocardiography
TV: Tricuspid valve
VHD: Valvular heart disease
